# Meta‐analysis of the impact of postoperative complications on survival after oesophagectomy for cancer

**DOI:** 10.1002/bjs5.64

**Published:** 2018-04-19

**Authors:** E. Booka, H. Takeuchi, K. Suda, K. Fukuda, R. Nakamura, N. Wada, H. Kawakubo, Y. Kitagawa

**Affiliations:** ^1^ Department of Surgery Keio University School of Medicine Tokyo Shizuoka Japan; ^2^ Department of Surgery Hamamatsu University School of Medicine Hamamatsu Shizuoka Japan

## Abstract

**Background:**

Oesophagectomy has a high risk of postoperative morbidity. The impact of postoperative complications on overall survival of oesophageal cancer remains unclear. This meta‐analysis addressed the impact of complications on long‐term survival following oesophagectomy.

**Methods:**

A search of PubMed and Cochrane Library databases was undertaken for systematic review of papers published between January 1995 and August 2016 that analysed the relation between postoperative complications and long‐term survival. In the meta‐analysis, data were pooled. The main outcome was overall survival (OS). Secondary endpoints included disease‐free (DFS) and cancer‐specific (CSS) survival.

**Results:**

A total of 357 citations was reviewed; 21 studies comprising 11 368 patients were included in the analyses. Overall, postoperative complications were associated with significantly decreased 5‐year OS (hazard ratio (HR) 1·16, 95 per cent c.i. 1·06 to 1·26; P = 0·001) and 5‐year CSS (HR 1·27, 1·09 to 1·47; P = 0·002). Pulmonary complications were associated with decreased 5‐year OS (HR 1·37, 1·16 to 1·62; P < 0·001), CSS (HR 1·60, 1·35 to 1·89; P < 0·001) and 5‐year DFS (HR 1·16, 1·00 to 1·33; P = 0·05). Patients with anastomotic leakage had significantly decreased 5‐year OS (HR 1·20, 1·10 to 1·30; P < 0·001), 5‐year CSS (HR 1·81, 1·11 to 2·95; P = 0·02) and 5‐year DFS (HR 1·13, 1·02 to 1·25; P = 0·01).

**Conclusion:**

Postoperative complications after oesophagectomy, including pulmonary complications and anastomotic leakage, decreased long‐term survival.

## Introduction

Worldwide, oesophageal cancer is the fifth most common cause of cancer‐related death in men, and the eighth in women^1^. The postoperative 5‐year survival rate in patients with AJCC stage I oesophageal cancer is approximately 90 per cent, and decreases to 45, 20 and 10 per cent in patients with stage II, III and IV disease respectively^2^ ^3^. For most patients without distant metastases, oesophagectomy is still the mainstay of cancer treatment with or without chemoradiotherapy^4^. Despite advances in surgical techniques and perioperative management^5^, oesophagectomy is a highly invasive procedure associated with serious postoperative complications^3^. In a Japanese national database comprising 5354 patients who underwent oesophagectomy in 2011 in 713 hospitals, the overall morbidity rate was 41·9 per cent, and 30‐day and surgery‐related mortality rates were 1·2 and 3·4 per cent respectively^6^.

The impact of postoperative complications on long‐term survival has been investigated for many cancers^3^ ^7^, ^8^. In some studies^3^ ^9^, ^10^, ^11^, ^12^, ^13^, ^14^, ^15^, ^16^, ^17^, ^18^, ^19^, ^20^, ^21^, a negative impact of complications following oesophagectomy on long‐term survival was reported. In other studies^3^ ^18^, ^19^ ^22^, ^23^, ^24^, ^25^, ^26^, ^27^, ^28^, ^29^, complications did not affect long‐term survival. Meta‐analyses focusing on the long‐term impact of postoperative complications are not available. A systematic review and meta‐analysis was therefore performed to assess the impact of postoperative complications on long‐term survival after oesophagectomy.

## Methods

A systematic review and meta‐analysis was carried out in accordance with the MOOSE criteria^30^. The key clinical question was: ‘Do postoperative complications after oesophagectomy for oesophageal cancer impact survival?’. A systematic literature search of studies describing clinical trials published from January 1995 to August 2016 was conducted. Literature searches of the PubMed and Cochrane Library databases were conducted using the search formula: (‘esophageal cancer’ OR ‘esophageal neoplasms’ OR ‘esophageal squamous cell carcinoma’) AND (‘esophagectomy’ OR ‘resection’ OR ‘surgery’) AND (‘anastomotic leakage’ OR ‘lung disease’ OR ‘pneumonia’ OR ‘postoperative complications’ OR ‘postoperative morbidity’ OR ‘pulmonary complications’ OR ‘respiratory tract disease’) AND (‘survival’ OR ‘disease free survival’ OR ‘mortality’ OR ‘prognosis’ OR ‘hospital mortality’ OR ‘neoplasm recurrence’).

### Eligibility criteria

RCTs and observational studies, including all types of operation (such as salvage surgery) and all types of neoadjuvant or adjuvant therapy, comparing the long‐term survival of patients with or without postoperative oesophagectomy complications were eligible for inclusion. Postoperative pulmonary complications, anastomotic leakage and the total number of postoperative oesophagectomy complications were included in the analysis. Other complications such as recurrent laryngeal nerve paralysis or atrial fibrillation were excluded. Articles for which the full text was not available in English were excluded.

### Data extraction

Data were extracted by one author and one reviewer from the Japan Medical Library Association. Any discrepancies were dealt with by discussion among all authors until consensus was reached. The primary outcome was 5‐year overall survival (OS) and secondary outcomes included disease‐free (DFS) and cancer‐specific (CSS) survival rates, which were extracted from the Kaplan–Meier curves in each study. The GRADE guidelines^31^ were used to evaluate the quality of individual studies, considering risk of bias, inconsistency, indirectness, imprecision, publication bias, size of effect, dose‐dependent gradient and plausible confounders. Studies assessed as of high quality in GRADE were included in the qualitative synthesis. It was expected that some studies would and others would not have included postoperative mortality. If some studies including postoperative mortality were excluded from the meta‐analysis, the sample size for each comparison would have been smaller, and the results would have been meaningless; these studies were therefore included in the meta‐analysis.

### Statistical analysis

Analyses were performed using Review Manager^®^ version 5.3 software (The Cochrane Collaboration, Oxford, UK). Pooled analysis was performed using a Mantel–Haenszel model, and the values were reported as hazard ratios (HRs) with 95 per cent confidence intervals. The significance of pooled HRs was determined by the *Z* test. *P* < 0·050 was considered statistically significant.

Statistical heterogeneity for each pooled estimate was assessed using Cochran's χ^2^ statistic and quantified with the *I* ^2^ statistic. An *I* ^2^ value exceeding 50 per cent was considered to indicate heterogeneity. When heterogeneity was detected, a random‐effects model was adopted; when heterogeneity was not observed, a fixed‐effect model was used.

## Results

### Search results

**Figure 1:**
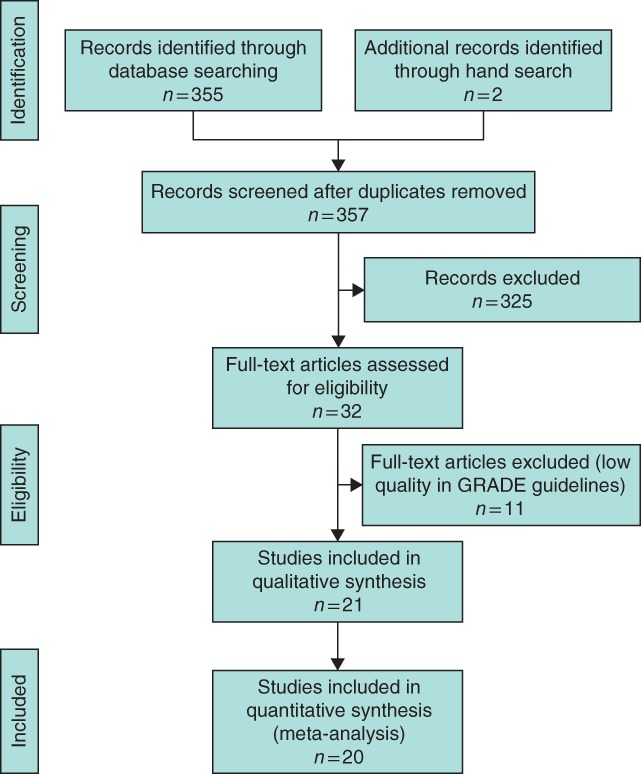
Flow diagram of inclusion and exclusion criteria for the study

A total of 357 potentially relevant studies were identified, of which 32 were eligible for full‐text review (*Fig*. 1). Twenty‐one studies^3^ ^9^, ^10^, ^11^, ^12^, ^13^, ^14^, ^15^, ^16^, ^17^, ^18^, ^19^, ^20^, ^21^ ^23^, ^24^, ^25^, ^26^, ^27^, ^28^, ^29^ met the eligibility criteria for qualitative synthesis (*Table* 1), and 20^3,9,10,12–21,23–29^ were eventually included for quantitative synthesis. The study that did not report 5‐year outcome results was excluded from the quantitative synthesis^11^. One study^19^ was a randomized trial; the others were observational studies.

**Table 1:** Characteristics of included studies

Reference	Year	Complication	No. with complications	No. without complications
Hirai et al. 12	1998	Any	47	100
Kinugasa et al. 9	2004	Pulmonary	38	80
Rizk et al. 10	2004	Any	138	372
Abou‐Jawde et al. 13	2005	Pulmonary	18	123
Junemann‐Ramirez et al. 23	2005	Anastomotic leak	9	251
Martin et al. 28	2005	Anastomotic leak	30	446
Ancona et al. 24	2006	Pulmonary	110	327
		Any	85	437
Ferri et al. 26	2006	Any	98	336
Lerut et al. 11	2009	Any	97	41
Hu et al. 14	2010	Any	90	271
D'Annoville et al. 25	2012	Pulmonary	118	223
Xia et al. 29	2013	Any	72	99
Lindner et al. 27	2014			
Adenocarcinoma		Any	14	49
Squamous cell carcinoma		Any	7	15
Booka et al. 3	2015	Pulmonary	64	220
		Anastomotic leak	55	229
Markar et al. 15	2015	Anastomotic leak	208	2231
Doorakkers et al. 16	2015	Any	75	221
Luc et al. 17	2015	Any	16	95
Baba et al. 18	2016	Pulmonary	99	403
		Any	217	285
Yamashita et al. 21	2016	Pulmonary	22	233
		Anastomotic leak	6	249
		Any	104	151
Kataoka et al. 19	2017	Pulmonary	22	130
		Anastomotic leak	21	131
Saeki et al. 20	2017			
Stage 0–2		Pulmonary	44	360
		Anastomotic leak	88	316
Stage 3–4		Pulmonary	15	161
		Anastomotic leak	26	150
Stage 0–4		Any	154	426

The severity of postoperative complications was based on each study, and there was variability on grading of the severities. Almost all studies categorized the severity of postoperative complications using the Clavien–Dindo classification^32^.

The range in 1‐, 3‐ and 5‐year OS rates for patients with complications was 47–84 per cent^27^ ^29^, 18–84 per cent^27^ ^29^ and 8–84 per cent^27^ ^29^ respectively. For patients without complications, the respective rates were 70–90 per cent^18^ ^29^, 30–71 per cent^18^ ^29^ and 10–66 per cent^27^ ^29^. The range in 1‐, 3‐, and 5‐year OS rates for patients with pulmonary complications was 28–87 per cent^13^ ^19^, 22–59 per cent^13^ ^19^ and 6–41 per cent^13^ ^20^ respectively, compared with 58–96 per cent^13^ ^19^, 36–78 per cent^13^ ^20^ and 29–65·7 per cent^13^ ^20^ for those without pulmonary complications. The range in 1‐, 3‐ and 5‐year OS rates for patients with anastomotic leakage was 66–86 per cent^19^ ^23^, 20–58 per cent^20^ and 15–57 per cent^19^ ^20^ respectively, compared with 64–94 per cent^19^ ^23^, 30–79 per cent^20^ ^23^ and 24–68 per cent^20^ ^23^ for those without anastomotic leakage. Of the 20 studies included in the quantitative synthesis, seven^15^, ^16^, ^17^ ^20^, ^23^ ^25^, ^27^ excluded and 13^3,9,10,12–14,18,19,21,24,26,28,29^ included perioperative mortality.

### Impact of pulmonary complications on survival

**Figure 2:**
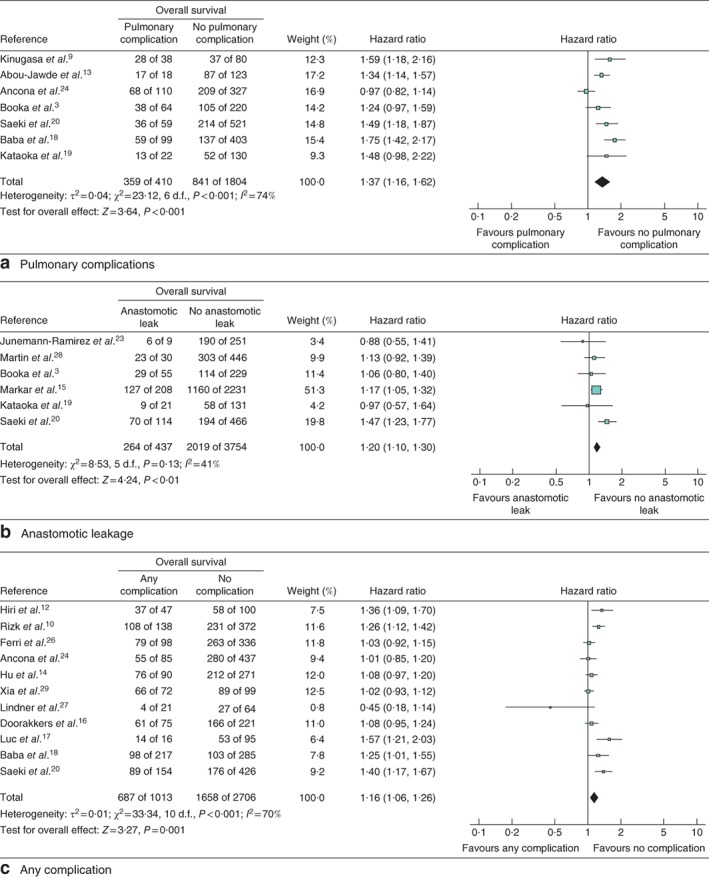
Forest plot comparing overall survival following oesophagectomy in patients with and without **a** pulmonary complications, **b** anastomotic leakage and **c** any complication. Mantel–Haenszel random‐effects (**a**,**c**) or fixed‐effect (**b**) models were used for meta‐analysis. Hazard ratios are shown with 95 per cent confidence intervals

**Figure 3:**
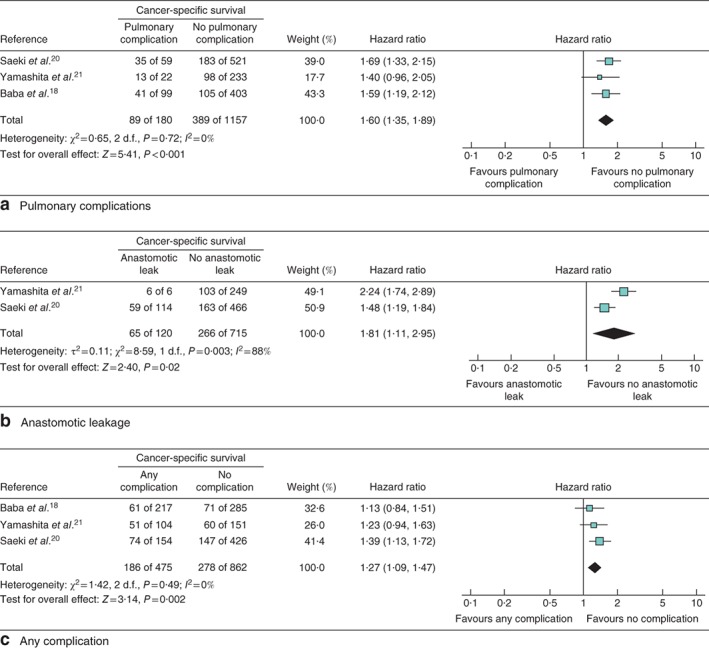
Forest plot comparing cancer‐specific survival following oesophagectomy in patients with and without **a** pulmonary complications, **b** anastomotic leakage and **c** any complication. Mantel–Haenszel fixed‐effect (**a**,**c**) or random‐effects (**b**) models were used for meta‐analysis. Hazard ratios are shown with 95 per cent confidence intervals

**Figure 4:**
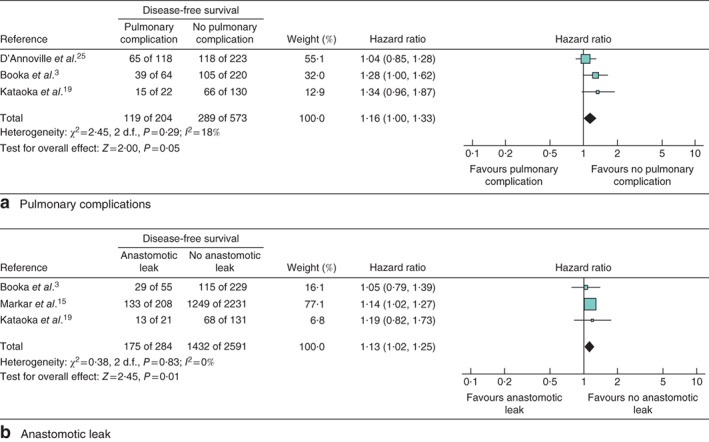
Forest plot comparing disease‐free survival following oesophagectomy in patients with and without **a** pulmonary complications and **b** anastomotic leakage. A Mantel–Haenszel fixed‐effect model was used for meta‐analysis. Hazard ratios are shown with 95 per cent confidence intervals

The impact of pulmonary complication on OS, CSS and DFS was evaluated in seven studies^3^ ^9^, ^13^ ^18^, ^19^, ^20^ ^24^ including 2214 patients (Fig. 2 a), three studies^18^ ^20^, ^21^ including 1337 patients (Fig. 3 a) and three studies^3^ ^19^, ^25^ including 777 patients (Fig. 4 a) respectively. Patients with pulmonary complications had significantly decreased 5‐year OS (HR 1·37, 95 per cent c.i. 1·16 to 1·62; P < 0·001), 5‐year CSS (HR 1·60, 1·35 to 1·89; P < 0·001) and 5‐year DFS (HR 1·16, 1·00 to 1·33; P = 0·05).

### Impact of anastomotic leakage on survival

The impact of anastomotic leakage on OS, CSS and DFS was evaluated in six studies^3^ ^15^, ^19^ ^20^, ^23^ ^28^ including 4191 patients (*Fig*. 2 *b*), two studies^20^ ^21^ including 835 patients (*Fig*. 3 *b*) and three studies^3^ ^15^, ^19^ including 2875 patients (*Fig*. 4 *b*) respectively. Patients with anastomotic leakage had significantly decreased 5‐year OS (HR 1·20, 95 per cent c.i. 1·10 to 1·30; *P* < 0·001), 5‐year CSS (HR 1·81, 1·11 to 2·95; *P* = 0·02) and 5‐year DFS (HR 1·13, 1·02 to 1·25; *P* = 0·01).

### Impact of overall complications on survival

The impact of postoperative complications in general on OS and CSS was evaluated in 11 studies^10^ ^12^, ^14^ ^16^, ^17^, ^18^ ^20^, ^24^ ^26^, ^27^ ^29^ including 3719 patients (*Fig*. 2 *c*) and three studies^18^ ^20^, ^21^ including 1337 patients (*Fig*. 3 *c*) respectively. There was no study investigating the impact of overall postoperative complications on DFS. Patients with more complications had significantly worse 5‐year OS (HR 1·16, 95 per cent c.i. 1·06 to 1·26; *P* = 0·001) and 5‐year CSS (HR 1·27, 1·09 to 1·47; *P* = 0·002).

### Impact of postoperative oesophagectomy complications on type of recurrence

Of the 21 eligible studies, four^12^ ^15^, ^17^ ^21^ investigated the impact of postoperative complications on recurrence type. One^15^ of these studies investigated the impact of anastomotic leakage on recurrence type and found it to be independently associated with locoregional and mixed recurrence (simultaneous local and distant recurrence) but not distant recurrence. The other three studies^12^ ^17^, ^21^ investigated the impact of overall complications on recurrence pattern. Meta‐analysis revealed that postoperative oesophagectomy complications did not specifically influence the site of recurrence (*Fig. S1*, supporting information).

### Risk of bias

Only studies that were assessed as high quality in GRADE^31^ were included. The *I* ^2^ statistic detected heterogeneities in the studies analysed in *Figs* 2 *a,* 2 *c* and 3 *b*; however, the forest plots showed that the direction of the point estimates was, in general, similar for all of the figures. As the heterogeneity was not significant, a random‐effects analysis was used, which resolved the heterogeneity that could not readily be explained, leading to reliable results.

## Discussion

In this meta‐analysis, postoperative complications after oesophagectomy had a significant negative impact on survival. Previous reports^3^ ^9^, ^10^, ^11^, ^12^, ^13^, ^14^, ^15^, ^16^, ^17^, ^18^, ^19^, ^20^, ^21^, ^22^, ^23^, ^24^, ^25^, ^26^, ^27^, ^28^, ^29^ of the impact of postoperative complications on long‐term survival have been inconsistent.

Long‐term survival was influenced in two ways. Some complications resulted in perioperative mortality, and there was an incremental effect of any type of complication on long‐term survival. Deterioration of the general condition may have affected long‐term OS and may have increased deaths unrelated to the oesophageal cancer^3^ ^19^. Moreover, worsening of the general condition may have led to delay or cessation of additional therapy after oesophagectomy, resulting in oesophageal cancer recurrence and having a negative impact on CSS and DFS^3^ ^19^.

Specific complications studied included pulmonary complications and anastomotic leakage. These often lead to generalized infection, which impacts significantly on the immunological system and in turn may lead to oesophageal cancer recurrence^3^. It was reported previously^33^ that infectious postoperative oesophagectomy complications significantly increased the levels of inflammatory cytokines such as interleukin (IL) 6 and IL‐8. Increased expression of both IL‐8 and its receptor CXCR‐2 have been correlated with tumour progression after oesophagectomy^34^. Anastomotic leakage may result in the local spread of viable tumour cells from stapled or sutured anastomoses. Locoregional recurrence after anastomotic leakage may be associated with a proinflammatory response that promotes tumour growth^15^.

Preventing postoperative complications may improve long‐term survival after oesophagectomy. High‐volume institutions with appropriate infrastructure are more able to deliver high‐quality outcomes^35^, ^36^, ^37^. Recently, minimally invasive oesophagectomy has become widespread, and may reduce the number of postoperative complications^38^. Moreover, better selection for surgery using risk models may improve outcomes^6^. Previously it was reported^3^ that oesophagectomy was not recommended for patients over 65 years of age or those with stage I if they were smokers. Definitive chemoradiotherapy may be recommended as an effective treatment for patients at high risk of postoperative surgical morbidity^4^ ^39^.

Survival was clearly affected by a higher mortality rate in patients with postoperative complications compared with that in patients without complications. The exclusion of postoperative mortality could have avoided this bias, but the sample size would have been significantly smaller. In the 13 studies^3^ ^9^, ^10^ ^12^, ^13^, ^14^ ^18^, ^19^ ^21^, ^24^ ^26^, ^28^ ^29^ that included postoperative mortality, however, the postoperative mortality rate was low. Thus the impact of postoperative mortality on long‐term survival was relatively limited and a superimposed effect of complications in the long‐term is clear.

This meta‐analysis had some limitations. Nearly all studies were retrospective and only one^19^ evaluated prospectively collected data. However, only the high‐quality observational studies were included in the meta‐analysis, and heterogeneity was overcome by using a random‐effects analysis. The severity of postoperative complications, neoadjuvant chemotherapy regimen and surgical procedure differed between studies. Some studies included neoadjuvant chemoradiotherapy for more advanced cancer stages or salvage surgery: factors known to be related to respiratory and gastric tube complications, and associated with poor survival^40^. The extent of confounding as a result of co‐morbidity and (neo)adjuvant treatments means that the present results should be interpreted with caution.

## Supporting information


**Fig. S1.** Forest plot comparing **A** locoregional, **B** lymphatic and **C** disseminated recurrence following oesophagectomy in patients with (+) and without (−) any complication. Mantel–Haenszel fixed‐effect (**A**) and random‐effects (**B,C)** models were used for meta‐analysis. Hazard ratios are shown with 95 per cent confidence intervals
