# Supplementary material for: Meta‐analysis of the impact of postoperative complications on survival after oesophagectomy for cancer
Source: BJS Open. 2018 Apr 19;2(5):276–84. doi: 10.1002/bjs5.64 (PMC6156161; doi:10.1002/bjs5.64)
Supplement: Supplementary file 1 — Fig. S1. Forest plot comparing A locoregional, B lymphatic and C disseminated recurrence following oesophagectomy in patients with (+) and without (−) any complication. Mantel–Haenszel fixed‐effect (A) and random‐effects (B,C) models were used for meta‐analysis. Hazard ratios are shown with 95 per cent confidence intervals [file BJS5-2-276-s001.docx]

**BJS5_64**

**Meta-analysis of the impact of postoperative complications on survival after oesophagectomy for cancer**

**E. Booka, H. Takeuchi, K. Suda, K. Fukuda, R. Nakamura, N. Wada, H. Kawakubo and Y. Kitagawa**


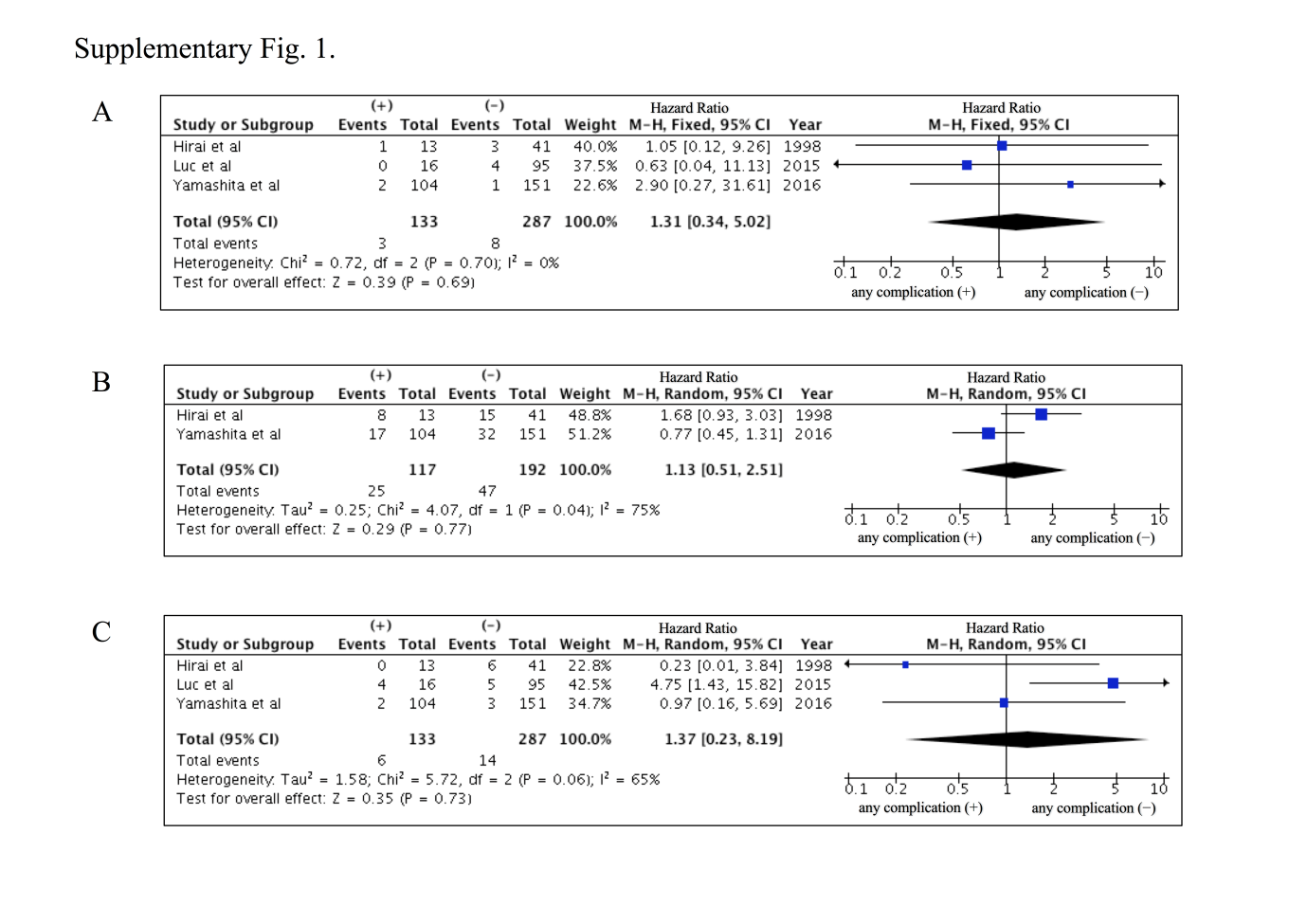


**Fig. S1** Forest plot comparing **A** locoregional, **B** lymphatic and **C** disseminated recurrence following oesophagectomy in patients with (+) and without (−) any complication. Mantel–Haenszel fixed-effect (**A**) and random-effects (**B,C)** models were used for meta-analysis. Hazard ratios are shown with 95 per cent confidence intervals
